# Predictors of Full Enteral Feeding Achievement in Very Low Birth Weight Infants

**DOI:** 10.1371/journal.pone.0092235

**Published:** 2014-03-19

**Authors:** Luigi Corvaglia, Maria Pia Fantini, Arianna Aceti, Dino Gibertoni, Paola Rucci, Dante Baronciani, Giacomo Faldella, on behalf of the “Emilia Romagna Perinatal Network”

**Affiliations:** 1 Neonatology and Neonatal Intensive Care Unit, S. Orsola-Malpighi Hospital, University of Bologna, Bologna, Italy; 2 Department of Biomedical and Neuromotor Sciences, University of Bologna, Bologna, Italy; 3 Servizio Presidi Ospedalieri, Direzione Generale Sanità e Politiche Sociali, Regione Emilia-Romagna, Bologna, Italy; University of East Anglia, United Kingdom

## Abstract

**Background:**

To elucidate the role of prenatal, neonatal and early postnatal variables in influencing the achievement of full enteral feeding (FEF) in very low birth weight (VLBW) infants and to determine whether neonatal intensive care units (NICUs) differ in this outcome.

**Methods:**

Population-based retrospective cohort study using data on 1,864 VLBW infants drawn from the “Emilia-Romagna Perinatal Network” Registry from 2004 to 2009. The outcome of interest was time to FEF achievement. Eleven prenatal, neonatal and early postnatal variables and the study NICUs were selected as potential predictors of time to FEF. Parametric survival analysis was used to model time to FEF as a function of the predictors. Marginal effects were used to obtain adjusted estimates of median time to FEF for specific subgroups of infants.

**Results:**

Lower gestational age, exclusive formula feeding, higher CRIB II score, maternal hypertension, cesarean delivery, SGA and PDA predicted delayed FEF. NICUs proved to be heterogeneous in terms of FEF achievement. Newborns with PDA had a 4.2 days longer predicted median time to FEF compared to those without PDA; newborns exclusively formula-fed had a 1.4 days longer time to FEF compared to those fed human milk.

**Conclusions:**

The results of our study suggest that time to FEF is influenced by clinical variables and NICU-specific practices. Knowledge of the variables associated with delayed/earlier FEF achievement could help in improving specific aspects of routine clinical management of VLBW infants and to reduce practice variability.

## Introduction

The advances in perinatal care over the last two decades have led to an improvement of neonatal survival worldwide [1], [2]. The increased survival of preterm infants is a challenge for the neonatologist, because preterm birth is known to be associated with short and long term morbidities [3]: preterm infants, especially those with very low birth weight (birth weight<1500 g, VLBW), often need active resuscitation at birth and intensive care in the neonatal period [1] and are at increased risk of early and late adverse outcome, including neurodevelopmental disabilities, respiratory, renal and cardiovascular problems, and features of the metabolic syndrome [4]–[6]. Quite interestingly, the risk of later impaired development applies to all preterm infants and seems to affect also infants who did not experience obvious neurological deficits during the neonatal period [7].

Optimal care provided during the neonatal period has the potential to reduce the risk of short and long term adverse outcome [8]. In this perspective, the balance between adequate nutrition and its possible complications is critical for preterm infants. Adequate parenteral and enteral nutrition is associated with better outcomes for preterm infants [8]. Actually, it has been demonstrated that nutrition during critical time windows in fetal and early life can affect permanently long-term health, including cardiovascular health [9] and neurodevelopment [10]. Early introduction and rapid achievement of full enteral feeding (FEF) is a priority in the nutritional management of preterm infants, because it reduces the need for central venous catheters (CVCs) and thus the risk of infection [11], and also reduces the length of hospital stay [12]. Furthermore, human milk (HM) feeding is thought to prevent early death, sepsis and necrotizing enterocolitis (NEC) [13], which is one of the most devastating diseases for preterm infants [14]. On the contrary, a delay in the introduction of progressive enteral feeding for VLBW infants has potential disadvantages, related to the impairment of the functional adaptation of the gastrointestinal (GI) tract [15], [16].

The need to attain FEF rapidly often conflicts with the physiologic immaturity of the GI function of preterm infants and also with the occurrence of various comorbidities in the neonatal period. In this perspective, it is extremely important to investigate risk factors occurring in the early neonatal period which influence the achievement of FEF, in order to optimize the nutritional management of VLBW infants and reduce the potential for several complications. Consideration of the clinical variables associated with delayed/earlier FEF achievement could help in ameliorating specific aspects of routine clinical management of VLBW infants.

The aim of the present study is to elucidate the role that some prenatal, neonatal and early postnatal variables play in favoring or delaying the achievement of FEF in VLBW infants and to determine whether neonatal intensive care units (NICUs) differ in this outcome, using data from a Regional Italian Registry.

## Methods

### Study population

The study was carried out in conformity with the Italian law on privacy and the regulations on data management of the Regional Health Authority. As anonymized administrative data are used routinely for health-care management, the study was exempt from notification to the Ethics Committee and no specific written informed consent was required to use patients' information.

Data were drawn from the “Emilia-Romagna Perinatal Network” Registry [17], a project sponsored by the Emilia-Romagna Health Agency, which is the result of a collaborative network of neonatologists and obstetricians. Emilia-Romagna is a region of North-Eastern Italy with a population of 4,342,135 inhabitants (Census 2011) [18]. The Registry database includes live-born infants with birth weight (BW) between 500 and 1500 grams admitted to the nine Level-3 NICUs of the Region.

Starting from 2002, data on live-born infants were collected prospectively from admission until discharge or death; data collection was continued for infants who were transferred to other hospitals. The database was designed according to the format of the Vermont Oxford Network Registry (VON) [19]; it included clinical characteristics of the infants, treatments received during hospitalization and, in addition, a section with obstetrical history. Demographic characteristics of the mothers and information on parity, obstetric history and index pregnancy were obtained through record linkage with birth certificates.

The study population included 2,441 VLBW infants born from January 2004 to December 2009 (1.0% of the 241,507 infants born in Emilia-Romagna in the same period). Newborns were excluded if they had congenital malformations, died on the first day of hospitalization or died subsequently but never received enteral feeding because of the severity of their clinical conditions. Records with missing or wrong data on all the predictors, as well as extreme outliers on hospital stay (>365 days) and time to FEF (>160 days), were also excluded.

The outcome of interest was time to FEF achievement, measured as the difference in days between the date when FEF was established and the birth date. FEF was defined as enteral tolerance of at least 150 ml/kg/day of milk.

For the purpose of the present study, on the basis of their clinical relevance and evidence from the literature, eleven prenatal, neonatal and early postnatal variables were selected for the analysis. NICUs were also included to examine the possible effect of different feeding practices on outcome. Because our aim was to identify early life predictors of time to FEF achievement, variables related to complications occurring later during hospitalization (such as retinopathy of prematurity [ROP], late-onset sepsis [LOS], and bronchopulmonary dysplasia [BPD]) were not considered.

The variables and the rationale for their inclusion in the analysis are detailed below:

### Prenatal variables

- Maternal pregnancy-induced hypertension, defined as pre-eclampsia and eclampsia: women with pre-eclampsia have higher risk of having children born preterm, with low birth weight or small for gestational age (SGA) [20]. Furthermore, infants born to pre-eclamptic mothers are at higher risk of feeding problems and increased hospital stay [12].
- Chorioamnionitis, which has been linked to several neonatal outcomes, including respiratory distress syndrome, for which chorioamnionitis seems to be protective, whereas the effect on intraventricular hemorrhage (IVH) and BPD appears to be detrimental [21]. The influence of chorioamnionitis on feeding tolerance has not been extensively investigated: however, chorioamnionitis can disturb fetal GI development [22] and antenatal inflammation can impair GI function, leading to NEC [23].
- Antenatal steroids prophylaxis, defined as complete (two doses) or incomplete [19]. Antenatal steroids administration induces organ maturation, especially of the lung [24]. Furthermore, it can influence gut permeability [25] and the secretion of gut peptides [26].

### Neonatal variables

- Mode of delivery, defined as vaginal delivery (VD), which included spontaneous, induced and instrumental delivery, and delivery by cesarean section (CS). Infants born by CS have significantly different physiology at birth compared to those born by VD, including altered feeding and metabolism [27]. Furthermore, gut flora in infants born by CS is different from that of infants born by VD [28], and this can have implications in terms of feeding tolerance and GI function [27].
- Gestational age (GA), as reported by the mother. If the difference between self-reported GA and GA calculated from early prenatal ultrasound examination was >1 week, the latter was used.
- Weight for GA. Infants were defined as appropriate, small and large for GA (AGA = 10°–90°, SGA<10°, and LGA>90° centile, respectively) according to neonatal growth charts developed *ad hoc* for the Italian population of preterm infants [29]. In the statistical analyses, a binary variable coded as SGA vs. AGA/LGA was used. Being small for gestational age is known to be associated with a worse outcome in extremely preterm infants [30].
- Sex. It has been demonstrated that, even with the advances in neonatal care, male preterm infants have higher mortality and poorer short- and long-term outcome than female counterparts [3], [31].

### Postnatal variables

- Clinical Risk Index for Babies (CRIB) II: CRIB II consists in a five-item score including GA, BW, sex, temperature on admission, and base excess; it provides a valid and simple method of risk-adjustment for neonatal intensive care [32]. The total score ranges from 0 to 22, with higher scores denoting a higher severity. This score, which has to be assessed within the first hour of life, can be considered as a proxy for the severity of the infant's clinical condition at birth.
- Early onset sepsis (EOS), diagnosed with a positive blood culture in the first seven days of life.
- Patent *ductus arteriosus* (PDA), assessed by ECHO in the first week of life. The presence of PDA and its treatment can influence tissue oxygenation, especially in the mesenteric region [33], and this can lead to impaired feeding tolerance.
- Feeding, defined as HM or formula-feeding during hospital stay. Specifically, HM feeding was defined as the use any own mother's or donor milk during hospital stay.

#### Characteristics of the NICUs

A questionnaire was administered to the heads of the nine NICUs to collect more detailed information on feeding practices in different GA/BW groups and in relation to the newborns' clinical conditions, in order to give a qualitative description of the possible sources of variability of the outcome among the NICUs. Practices regarding PDA management were also collected.

### Statistical analysis

Newborns' characteristics were compared among the study NICUs using ANOVA or chi-square test as appropriate. Following significant test, post-hoc pairwise comparisons were carried out using Bonferroni correction to the probability level.

The study outcome was time (in days) to FEF achievement. A parametric survival analysis was used to estimate the relationship of prenatal, neonatal and early postnatal variables with time to FEF. The main advantages of this analytical approach are the use of information provided by each individual in the cohort (including, as censored at the date of death, those who died after the first day of hospitalization without achieving FEF) and the use of an appropriate distribution for time to FEF estimation, reflecting the observed temporal pattern of occurrence of this outcome.

In this study we chose a log-logistic hazard function [34] to take into account that the frequency distribution of time to FEF achievement was not constant over time but had a peak in the first 10 days of life. The relationship between a predictor and the outcome was provided in terms of time ratios (TR). A TR>1 indicated a longer time to FEF (a negative effect of the predictor), while a TR<1 indicated a shorter time to FEF (a positive effect of the predictor). A significant TR indicated that the null hypothesis of independence between the predictor and the outcome, expressed by TR = 1, could be rejected.

The nine participating NICUs were included in the survival model as eight dummy variables in order to take into account the possible effect of practice differences on time to FEF. NICU ‘B’ was chosen as the reference unit because it had the highest number of newborns.

The initial model included all the predictors identified *a priori* on a clinical basis. A backward elimination criterion was used to obtain the final model, removing one at a time those predictors whose effect on time to FEF was not significant (p>0.05).

Marginal effects were obtained for each variable using the estimates of time to FEF from the final model [35]. For instance, the marginal effect of the type of feeding was the difference in days to FEF achievement that would be expected if two subjects differed only for type of feeding. In order to examine in deeper detail the effect of PDA, which proved to be a strong predictor for time to FEF achievement, secondary survival analyses were carried out to compare this outcome among infants without PDA and those with PDA either untreated or receiving pharmacological and/or surgical treatment.

Statistical analyses were carried out using Stata version 13.0 (StataCorp LP, College Station, Texas, USA). Streg procedure was used for parametric survival analysis and margins procedure to estimate marginal effects.

## Results

Of the 2,441 newborns, 219 (9.0%) were excluded because they had congenital malformations, 58 (2.4%) because they died on their first day of hospitalization, and 59 (2.4%) because they never received enteral feeding. Among the 2,105 subjects eligible for the study, 234 (11.1%) were excluded due to missing or wrong data and 7 because they were extreme outliers (0.3%). Thus, 1,864 newborns were included in the survival analysis. Of these subjects, 1,775 (95.2%) achieved FEF; 109 (5.9%) died during hospitalization and, of these, 20 (18.4%) died after having reached FEF. Compared with newborns included in the survival analysis, those who were not included were significantly more likely to have been born by vaginal delivery at a lower GA, and to have a higher CRIB II score and a higher incidence of IVH. Moreover, they were less likely to have received antenatal steroids, to have been oxygen dependent at 36 weeks of corrected age, to have had periventricular leukomalacia and LOS, and have required UVC for fewer days.

The clinical characteristics of the sample are provided in Table 1. Fifteen point four percent of the mothers had hypertension during pregnancy. Mean GA was 29.1 weeks, 48.5% of the infants were males, 20.1% were SGA. Mean CRIB II score was 2.33; infants who received any HM during hospital stay were 70.8%. A diagnosis of PDA was made in 38.4% of the infants. The incidence of major complications was 2.1% for EOS, 4.0% for NEC, 15.1% for ROP (grade 1–2: 75.9%, grade 3–4: 24.1%), 5.1% for LOS, 6.0% for IVH and 12.4% for BPD.

**Table 1:** Clinical characteristics of the very low birth weight infants included in the study cohort (n = 1864).

VARIABLES	n (%)^a^	p-value for ANOVA or χ^2^ test	Post-hoc pairwise comparisons at p = 0.001
Pregnancy due to In Vitro Fertilization	189 (10.2)	0.307^b^	none
Maternal pregnancy-induced hypertension	287 (15.4)	0.001^b^	D>B
Prolonged rupture of membrane	359 (19.3)	<0.001^b^	AI>BG; E>B
Chorioamnionitis	56 (3.0)	<0.001^b^	A>BGI
Antenatal steroids prophylaxis		<0.001^b^	
Complete cycle	1166 (62.5)		A>CDEGHI
Incomplete cycle	283 (15.2)		A<BDEFHI
Not administered	416 (22.3)		G>ABF; F<DH
Multiple births	533 (28.6)	0.100^b^	none
Mode of delivery		0.019^b^	
Vaginal	258 (13.8)		B<C
Cesarean	1607 (86.2)		B>C
Gestational age, wks			
Mean, SD	29.1 (2.7)	<0.001^c^	G>AFH
<24	33 (1.8)		None
24–26	302 (16.2)		G<ACDEFHI
27–29	692 (37.1)		None
30–32	645 (34.6)		G>ACEFHI
>32	193 (10.4)		None
Small for gestational age	375 (20.1)	0.001^b^	C>AFH
Males	904 (48.5)	0.012^b^	H>A
CRIB II score			
Mean, SD	2.3 (2.9)	<0.001^c^	G<BH
Endotracheal intubation	576 (30.9)	<0.001^b^	G<ACEFH; I<CEF; B<CDE; D<E
Type of feeding		<0.001^b^	
Human or mixed	1321 (70.8)		F<ABCDEHI; C>EGI
Formula only	544 (29.2)		F>ABCDEHI, C<EGI
Umbilical venous catheter	1403 (75.2)		IAD>BCEFH; GB>CEFH; E>FH; C>F
Mean n. days (SD)	4.9 (4.1)	<0.001^c^	D>BEFGHI; A>EFH; HF<BCGI; E<BC
Early-onset sepsis	39 (2.1)	<0.001^b^	F>H
Patent *ductus arteriosus*	716 (38.4)	<0.001^b^	CG<ABDEFH; I<DEF; D>AH
Necrotizing enterocolitis	75 (4.0)	0.076^b^	None
Nasal continuous positive airway pressure	1357 (72.8)		C<ABDEFI; G<BEF; F>ADHI; E>DH; B>H
Mean n. days (SD)	5.9 (9.5)	<0.001^c^	AB>CGHI; E>CHI
Intraventricular hemorrhage	111 (6.0)	<0.001^b^	B<C
Oxygen dependency at 36 weeks	232 (12.4)	0.001^b^	B>F
Late-onset sepsis	95 (5.1)	0.001^b^	H<BCE
Retinopathy of prematurity	283 (15.2)	<0.001^b^	AI>BCDFGH; GH<BCE
Periventricular leukomalacia	248 (13.3)	<0.001^b^	F>ABCDGHI; E>ABCH; B<DGI; H<DI

a Unless otherwise specified.

b p-values for χ^2^ test.

c p-values for ANOVA test.

The median time for achieving FEF was 13 days (IQR 7-24 days) and the mean time 18.25 days (SD = 17.56). The nine NICUs differed significantly on time to FEF achievement (ANOVA test: F = 31.11, p<0.001) and on the frequency of several prenatal and postnatal variables (Table 1). Post-hoc pairwise comparisons found that one NICU (NICU G) was significantly different from the others in terms of gestational age of the admitted infants. This was due to the admission criteria used in this Unit, which admits only infants with GA higher than 27 weeks. This was likely to result in a lower clinical severity of newborns recruited in this unit compared to the others. The other significant post-hoc differences among NICUs were not systematic and could not be interpreted in terms of variability in organization or case mix.

**Figure 1:**
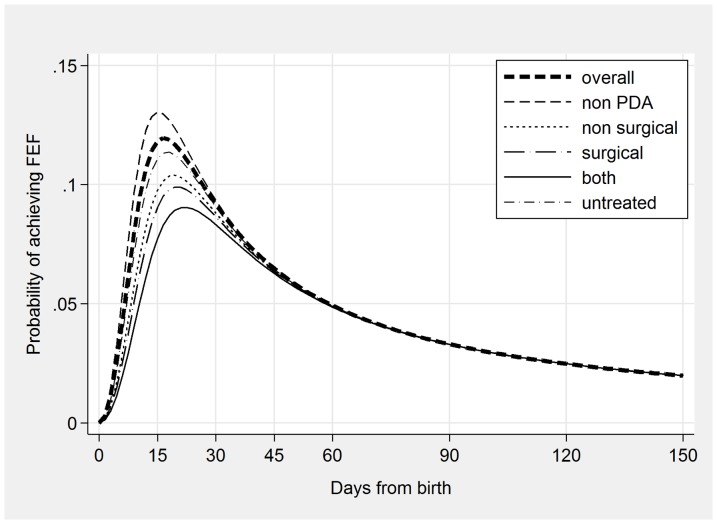
Hazard function of time to full enteral feeding achievement for all newborns (thick line) and for subgroups determined by presence and treatment of patent ductus arteriosus (PDA).

Starting from an initial survival model including all predictors, a final model was obtained including NICUs and seven predictors significantly associated with time to FEF achievement (Table 2). The hazard function (Figure 1) obtained with this model showed that the probability of achieving FEF had an initial steep increase, a peak around 15 days from birth (when the probability of achieving FEF was the highest), and a subsequent progressive decline. The estimated median time to FEF was 12.9 days (IQR = 8.0–21.5 days). Higher GA was associated with an earlier achievement of FEF (TR for GA was 0.841, indicating that, for each additional week of gestation, time to FEF was reduced by 15.9%). On the contrary, the other six predictors (SGA, PDA, formula feeding, mode of delivery, CRIB II and maternal hypertension) were associated with a significantly longer time to FEF. The TR for SGA was 1.166 and for PDA was 1.276, showing that time to FEF achievement was 16.6% longer in SGA infants compared with AGA and LGA infants, and 27.6% longer in infants with PDA compared with those without PDA. Maternal hypertension delayed time to FEF by 11.2%, and CS by 10.4% compared to VD, while each point increase in CRIB II score was associated with a 4.8% longer time to FEF. Being exclusively formula-fed increased time to FEF by 8.5%. Heterogeneity among the NICUs was very high: the TRs ranged from 0.359 to 1.948 and with only one exception they were significantly different from the reference NICU.

**Table 2:** Predictors of time to FEF.

	*Initial Model*		*Final Model*	
*Predictors*	*TR*	*p-value*	*TR*	*p-value*
Maternal hypertension (yes/no)	1.110 (1.030–1.197)	0.006	1.112 (1.032–1.199)	0.006
GA (weeks)	0.840 (0.825–0.854)	<0.001	0.841 (0.827–0.855)	<0.001
Weight for GA (SGA vs. AGA, LGA)	1.168 (1.070–1.274)	0.001	1.166 (1.066–1.272)	0.001
CRIB II score	1.047 (1.033–1.061)	<0.001	1.048 (1.034–1.062)	<0.001
PDA (yes/no)	1.273 (1.196–1.356)	<0.001	1.276 (1.198–1.358)	<0.001
Mode of delivery (caesarean vs. vaginal)	1.106 (1.016–1.205)	0.020	1.104 (1.015–1.201)	0.021
Formula milk fed	1.085 (1.020–1.153)	0.012	1.085 (1.021–1.153)	0.009
NICU A	1.590 (1.423–1.776)	<0.001	1.585 (1.420–1.768)	<0.001
NICU C	1.423 (1.280–1.583)	<0.001	1.426 (1.282–1.585)	<0.001
NICU D	1.946 (1.738–2.180)	<0.001	1.948 (1.740–2.180)	<0.001
NICU E	1.058 (0.955–1.172)	0.284	1.057 (0.955–1.171)	0.284
NICU F	0.815 (0.733–0.907)	<0.001	0.819 (0.737–0.911)	<0.001
NICU G	1.236 (1.072–1.425)	0.003	1.238 (1.075–1.426)	0.003
NICU H	0.360 (0.323–0.401)	<0.001	0.359 (0.323–0.400)	<0.001
NICU I	0.868 (0.778–0.968)	0.011	0.869 (0.779–0.970)	0.012
Chorioamnionitis (yes/no)	0.960 (0.815–1.132)	0.630	-	
Antenatal steroid prophylaxis (ref.: complete cycle)				
Incomplete cycle	1.016 (0.940–1.098)	0.685	-	
Not administered	1.022 (0.955–1.094)	0.525	-	
Sex (male vs. female)	0.989 (0.936–1.044)	0.684	-	
Early onset sepsis (yes/no)	1.097 (0.903–1.331)	0.351	-	
Constant	9.349 (8.287–10.548)	<0.001	9.365 (8.357–10.495)	<0.001
ln(gamma)	−1.080 (−1.119; −1.041)	<0.001	−1,080 (−1,119; −1.040)	<0.001

Results of the survival loglogistic model (N = 1864).

Correlations among predictors of the final model higher than 0.30 (absolute value) were found for GA with SGA, CRIB II score and PDA, and for CRIB II score with PDA. None of these correlations exceeded 0.63, suggesting that the predictors were not redundant in the model.

Marginal effects were higher for the variables which had a stronger effect on outcome (Table 3): infants with PDA had a 4.2 days longer time needed to achieve FEF than infants without PDA, while, for each additional week of GA, time to FEF was reduced by 3 days. Each one-point increase in CRIB II score contributed for a 0.8 days delay in achieving FEF: for instance, a newborn with a CRIB II score of 4 would achieve FEF 3.2 days after a newborn with a CRIB II score of 0. Exclusively formula-fed infants reached FEF 1.4 days later than infants fed HM.

**Table 3:** Marginal effects (differences in days) in predicted median time to full enteral feeding achievement for FEF predictors.

	*ME*	*Std. Err.*	*P*
Cesarean delivery	1.66	0.701	0.018
Maternal hypertension	1.91	0.714	0.008
GA	−3.00	0.168	<0.001
SGA	2.83	0.868	0.001
CRIB II	0.81	0.121	<0.001
Formula only	1.43	0.556	0.010
PDA	4.18	0.554	<0.001
NICU A	9.10	1.188	<0.001
NICU C	6.63	1.056	<0.001
NICU D	14.75	1.473	<0.001
NICU E	0.89	0.835	0.284
NICU F	−2.81	0.763	<0.001
NICU G	3.70	1.332	0.005
NICU H	−9.97	0.618	<0.001
NICU I	−2.03	0.805	0.012

Note: for instance, the first row indicates that for newborns born with cesarean delivery and equal on all the other variables it takes 1.66 more days to achieve FEF w.r.t. those born with vaginal delivery.

Remarkable differences among NICUs were found: for example, after adjusting for the other variables in the model, newborns in NICU D achieved FEF 14.7 days later than in the reference NICU, those from NICU A 9.1 days later, and those from NICU H 10 days earlier.

The secondary analysis carried out to examine the effect of PDA treatment showed that observed median time to FEF was 15.1 days in infants without PDA, and increased to 17.8 days in infants with PDA who did not receive any treatment, to 19.3 days in those receiving a pharmacological treatment, and to up to 21.7 days in those who underwent surgery in combination with a pharmacological treatment. These findings were confirmed by survival analysis: the hazard functions estimated for these subgroups (Figure 1) showed that infants without PDA had the highest probability to achieve FEF earlier, followed by infants who had PDA untreated or treated only pharmacologically. Infants receiving surgery (either alone or in combination with pharmacological treatment) had the latest FEF achievement.

### Characteristics of the NICUs

The survey, conducted to explore the different attitudes towards feeding VLBW infants, showed that the large majority of the NICUS (7 out of 9) had a standardized protocol for enteral feeding.

However, feeding practices were quite heterogeneous among NICUs. Furthermore, feeding protocols in each NICU were modulated according to different cut-offs (BW in 4 NICUs, GA in 2 NICUs, and a combination of BW and GA in the remaining 3 NICUs).

In smaller/younger infants, minimal enteral feeding (MEF) was used in all the NICUs, starting at 0–2 days in six NICUs, and at 3–5 days in the other three NICUs; the daily increase of enteral intake was less than 15 ml/kg/day in the majority of the NICUs (7/9). Reasons for feeding withdrawal were also heterogeneous, including bilious aspirates, red blood cells transfusions, and presence and treatment of PDA.

Clinical practices regarding PDA management were also examined: none of the NICUs used prophylactic PDA treatment, and all of them treated PDA only when hemodynamically significant. The first therapeutical approach to PDA was pharmacological for all the NICUs: 7 used exclusively Ibuprofen, 1 Ibuprofen for newborns less than 30 weeks GA and Indomethacin for newborns>30 weeks GA, and 1 used exclusively Indomethacin. Surgical treatment was a second-line treatment in all the NICUs, after the failure of two consecutive pharmacological courses.

## Discussion

The present study aimed to evaluate the effect of prenatal, neonatal and early postnatal conditions on time to FEF achievement in VLBW infants.

GA and PDA proved to be the strongest predictors of time to FEF. As expected, higher GA predicted an earlier achievement of FEF: specifically, each one-week increase in GA reduced time to FEF by 15.9% (estimated reduction of more than 3 days). To our knowledge, evidence linking GA to FEF achievement in VLBW infants is scanty [36], though the influence of GA on neonatal and long-term outcome (mortality and neurodevelopment) has been emphasized also in previous studies [1], [3].

In the present study, PDA increased time to FEF achievement by 27.6% (estimated delay of more than 4 days). The presence of a clinically significant PDA, especially when associated with sepsis, has been recognized as a risk factor for a delayed start of enteral feeding and FEF achievement [37]; the adverse effect of PDA on enteral feeding may be related to the impairment of mesenteric perfusion [33]. We estimated that the presence of PDA and its treatment may increase time to FEF up to about 7 days (from 2.7 days for newborns with untreated PDA to 6.6 days for the newborns whose PDA was treated pharmacologically and surgically). The delay in FEF achievement can be related to the direct influence of PDA on feeding tolerance: this hypothesis is supported by the reduction in NEC incidence, which was documented in extremely low birth weight infants following PDA prophylactic surgical ligation [38]. Furthermore, such a delay could be a direct consequence of a more prudent attitude of the clinician towards a progression to FEF in infants with PDA. Based on current evidence, there is no consensus on the optimal nutritional approach to VLBW infants with PDA. Future studies should investigate how FEF achievement could be optimized in VLBW infants with risk factors such as PDA.

Infants fed HM reached FEF slightly earlier than exclusively formula-fed infants, after adjusting for all the other newborns' characteristics. The estimated reduction of the time to FEF in HM-fed infants was 1.4 days. This reduction could be clinically significant, as results from cohort studies in extremely low birth weight newborns fed HM indicate that very early establishment of FEF with HM is associated with lower occurrence of complications such as LOS [39].

In our study population, SGA increased time to FEF achievement by 16.6%. Evidence from the literature suggests that IUGR infants have increased risk for several adverse neonatal outcomes [40]–[45]. The effect of being IUGR on FEF achievement is controversial and available data are scarce. One randomized controlled trial (RCT) comparing IUGR vs. non-IUGR infants in terms of feeding tolerance and FEF achievement found no differences between these two groups of VLBW infants [46]. The quality of prenatal data on intrauterine growth restriction in the Emilia Romagna Registry is poor; for this reason, IUGR was not included in the analysis. However, data from literature also suggest an effect of being SGA on FEF achievement: actually, a population-based study showed that SGA infants take significantly longer to achieve FEF than AGA counterparts [30]. Growth-restricted infants are at increased risk of developing NEC [47] and for this reason the start of enteral feeding is frequently delayed, even if it is unclear whether such a delay would be beneficial. A very recent RCT performed in preterm growth-restricted infants demonstrated that early feeding was not associated with a higher risk of NEC [48].

Our results on the association between FEF, GA, SGA and PDA are consistent with a recent study based on a retrospective cohort of preterm infants [49], in which the authors included as predictors also sepsis and NEC. The inclusion of these variables, however, may be misleading, because the association between achievement of FEF and these clinical outcomes does not have a clear direction. In other words, sepsis and NEC may occur before or after FEF achievement, and thus cannot be unambiguously considered as potential predictors of FEF.

An increase in CRIB II score was associated with a delay in FEF achievement. To the best of our knowledge, no previous studies assessed the role of this predictor on time to FEF in preterm infants. CRIB II score, which provides a recalibrated and simplified scoring system that avoids the potential problems of early treatment bias [32], has been used to date to predict mortality and neurodevelopmental outcome [50], [51].

Maternal hypertension increased time to FEF achievement by 11.2%. Previous studies have reported a similar association [12]. One explanation for this finding is that the abnormality of the blood flow *in utero* may persist in the splanchnic circulation for several days after birth, compromising feeding tolerance and FEF achievement [52]. Furthermore, it is likely that the presence of maternal hypertension determines a more cautious clinician's attitude towards introducing enteral feeding and thus a delayed achievement of FEF.

Infants born by CS reached FEF later than infants born by VD. We can speculate that this delay could be attributed to the alterations in gut microbiota which occur following CS [28], which have been linked to feeding intolerance in term newborns [27]. Furthermore, disturbances in the development of normal colonization patterns in the developing gut have been recently linked to an increased risk of NEC [53].

In addition, our results show that, after adjusting for the effect of other variables, NICUs proved to be still heterogeneous in time to FEF. This indicates that time to FEF was significantly accounted for by unmeasured NICUs characteristics, including differences in clinical practice or in the organizational set-up. The survey which was conducted in collaboration with the heads of the nine NICUs showed that most of the NICUs had a formal protocol for the nutrition of preterm infants, but these protocols were quite heterogeneous as well. The differences in clinical and nutritional practices may at least partially explain the significant variation in time to FEF among NICUs (up to almost 15 days). The identification of differences in clinical practices among NICUs could be the starting point for the development of clinical *audit* activities, aiming to identify “best clinical practices” and promote quality improvement in neonatal care.

One of the methodological strengths of the study is its design: the advantage of population-based studies over RCTs is that the former include data collected systematically from unselected populations. In particular, data obtained from large population-based registries, such as the VON [54], are specifically intended to assist clinicians in answering research questions, identifying opportunities for quality of care improvement and monitoring the success of their improvement efforts [55], [56]. Furthermore, another strength is the use of registry data, which were collected in a standardized way and entered using a web-based form. However, data quality was not completely satisfactory, as the proportion of missing or incorrect data was 18%. More importantly, this registry did not include detailed information on clinical aspects and practice of feeding, such as the type and amount of feeds and the starting date of enteral feeding. This precluded the possibility to test the effect of initiation of enteral feeds on time to FEF.

It has to be noted that, although our population included a slightly higher proportion of very preterm newborns compared to VON data (GA<27 weeks 18.0% vs. 16.1%), mortality was lower (14.9% vs. 16.6%), as well as the incidence of neonatal complications such as NEC (4.6% vs. 6.7%) [54]–[56]. However, even in this high quality of care setting, median time to FEF was approximately 13 days, with considerable variation among NICUs. Efforts should be made to reduce variability in feeding practices among NICU: actually, a long time to FEF implies the persistence of CVCs, and the consequent exposure to a high risk of complications [11].

In conclusion, the present study identifies some clinical predictors of the time to FEF achievement in VLBW infants. Knowledge of risk factors associated with FEF achievement could help in ameliorating specific aspects of routine clinical management of VLBW infants. Efforts are warranted to optimize nutritional management of VLBW infants and reduce NICU variability in feeding practices in order to avoid potential complications of a delayed achievement of FEF.
